# Polyamine metabolism in flax in response to treatment with pathogenic and non–pathogenic *Fusarium* strains

**DOI:** 10.3389/fpls.2015.00291

**Published:** 2015-04-29

**Authors:** Wioleta Wojtasik, Anna Kulma, Katarzyna Namysł, Marta Preisner, Jan Szopa

**Affiliations:** Faculty of Biotechnology, University of WrocławWrocław, Poland

**Keywords:** polyamines, pathogenic and non-pathogenic *Fusarium* strains, plant infection, defense mechanism, flax

## Abstract

Flax crop yield is limited by various environmental stress factors, but the largest crop losses worldwide are caused by *Fusarium* infection. Polyamines are one of the many plant metabolites possibly involved in the plant response to infection. However, in flax plants the polyamine composition, genes involved in polyamine synthesis, and in particular their regulation, were previously unknown. The aim of this study was to investigate the polyamine synthesis pathway in flax and its involvement in response to pathogen infection. It is well established that polyamines are essential for the growth and development of both plants and fungi, but their role in pathogen infection still remains unknown. In our study we correlated the expression of genes involved in polyamine metabolism with the polyamine levels in plant tissues and compared the results for flax seedlings treated with two pathogenic and one non-pathogenic strains of *Fusarium*. We observed an increase in the expression of genes participating in polyamine synthesis after fungal infection, and it was reflected in an increase of polyamine content in the plant tissues. The highest level of mRNA was characteristic for ornithine decarboxylase during infection with all tested, pathogenic and non-pathogenic, *Fusarium* strains and the arginine decarboxylase gene during infection with the pathogenic strain of *Fusarium culmorum*. The main polyamine identified in the flax seedlings was putrescine, and its level changed the most during infection. Moreover, the considerable increase in the contents of cell wall-bound polyamines compared to the levels of free and conjugated polyamines may indicate that their main role during pathogen infection lies in strengthening of the cell wall. *In vitro* experiments showed that the polyamines inhibit *Fusarium* growth, which suggests that they play an important role in plant defense mechanisms. Furthermore, changes in metabolism and content of polyamines indicate different defense mechanisms activated in flax in response to infection by pathogenic and non-pathogenic *Fusarium* strains.

## Introduction

Flax (*Linum usitatissimum* L.) is a valuable plant with a long history of cultivation. It is a source of oil and fibers used in the pharmaceutical, cosmetics, food, paper, and textile industries. The biggest advantage of flax is that the whole plant can be used, so it can be qualified as a no-waste, multipurpose plant. The competition with cotton as a source of fiber and rape as a source of oil caused a decrease in the cultivation of flax in recent years. However, the success of diversification and improvement of flax properties through genetic modifications gave rise to novel biomedical applications, reviving interest in the crop (Czemplik and Szopa, 2009). However, as with other flax plant products, the decline in productivity caused by pathogen infection alters the yield and quality of flax fibers (Henriksson et al., 1997).

Flax diseases are mainly caused by fungi from the genus *Fusarium*, infection with which can lead to over 20% loss in the flax yield. All strains of *Fusarium oxysporum* are saprophytic, and some of them are pathogenic to different species, inducing wilt and root rot and finally plant death. Non-pathogenic strains can colonize the surface of roots without causing disease or, similarly to pathogenic strains, they can penetrate roots, leading to the defense responses, but without the development of disease. In the case of non-pathogenic infection, plants are capable of stopping the fungal invasion by creating a defense barrier in the cortex, whereas pathogens try to avoid induction of the plant defense responses at this stage. Another difference is that pathogenic strains are able to induce a hypersensitive reaction (HR), while non-pathogenic strains do not have this ability (Olivain et al., 2003).

The most dangerous pathogen of flax is *F. oxysporum* sp. *lini.* An infection starts when fungi infiltrate into flax root cells, and advance intracellularly into the xylem by producing microconidia, which germinate and thus block the vascular vessels, preventing water and nutrient translocation. This leads to epinasty and yellowing of the lower leaves, progressive wilting, and eventually death. After the plant dies, the fungus invades all of the tissues, sporulates, and infects neighboring plants (Michielse and Rep, 2009). Another, less specific flax pathogen is *Fusarium culmorum*, which causes the common root rot and *Fusarium* head blight (FHB) in many plant species. The typical growth patterns of *F. culmorum* are accompanied by an increase in pH during infection followed by an increase in the activity of extracellular enzymes able to hydrolyse the cellulose, xylan, and pectin of the plant cell wall, which allows host tissue invasion within 3 or 4 days (Scherm et al., 2013). This causes shoots to darken and dry, with plants easily broken off at ground level, leading to plant death (Olivain et al., 2003; Berrocal-Lobo and Molina, 2008).

Many secondary metabolites are involved in plant resistance to pathogens, including phenolic acids, flavonoids, lignin, terpenoids, and polyamines. In flax, many of these metabolites have been identified, and their function in flax resistance to pathogens has been established. However, metabolism of polyamines in flax has not yet been described. Their possible involvement in a pathogen response was indicated by isolation of the biosynthetic gene arginine decarboxylase from a subtractive library of a *Fusarium*-infected flax seedling (Wojtasik et al., 2011; Kostyn et al., 2012).

Polyamines are positively charged aliphatic amines that are commonly found in all organisms and are responsible for various fundamental processes. Putrescine, spermidine and spermine occur either in a free form, or conjugated to phenolic acids, mainly to ferulic acid, coumaric acid, and caffeic acid (hydroxycinnamic acid amide derivatives), or bound with macromolecules, especially with DNA, RNA, proteins and other compounds of the plant cell wall, such as pectin (Walters, 2000).

**Figure 1:**
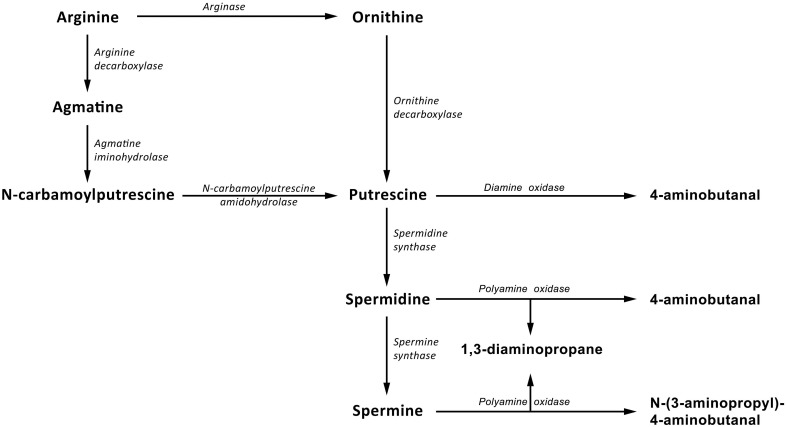
**Polyamine biosynthetic pathway in plants**. Putrescine is synthesized directly from ornithine by ornithine decarboxylase and indirectly from arginine by arginine decarboxylase, agmatine iminohydrolase and N-carbamoylputrescine amidohydrolase. Spermidine and spermine are, respectively, formed by the subsequent addition of an aminopropyl moiety to putrescine and spermidine by spermidine synthase and spermine synthase, respectively. Two enzymes, diamine oxidase, and polyamine oxidase, participate in degradation of polyamine.

There are two possible pathways of polyamine biosynthesis in plants (Figure 1). The first consists of the conversion of arginine into ornithine by arginase (ARG, EC 3.5.3.1). Then, putrescine is created through the reaction catalyzed by ornithine decarboxylase (ODC, EC 4.1.1.17). The other pathway requires several enzymes—arginine decarboxylase (ADC, EC 4.1.1.19), agmatine deiminase (AIH, EC 3.5.3.12) and N-carbamoylputrescine amidase (NCPAH, EC 3.5.1.53)—to produce putrescine. Thereafter, reactions catalyzed by spermidine synthase (SPDS, EC 2.5.1.16) and spermine synthase (SPMS, EC 2.5.1.22) cause consecutive aminopropyl groups to be attached to putrescine, giving spermidine, and spermine. While so many enzymes are involved in polyamine synthesis, only two known enzymes are involved in their degradation: diamine oxidase (DAO, EC 1.4.3.22) and polyamine oxidase (PAO, EC 1.5.3.14), which catalyze the oxidative deamination of putrescine, spermidine, and spermine (Kusano et al., 2007). The activity of both of those branches can differ between plant species. For example, there is a doubt whether the ornithine branch is functional in *Arabidopsis*, pointing to the importance of research on metabolic pathways not only in model plants but in particular in those of agricultural importance (Hanfrey et al., 2001; Tassoni et al., 2003).

The polyamine metabolic pathway has also been studied using transgenic plants. For example, overexpression of oat arginine decarboxylase caused a significant increase in ADC activity and accumulation of agmatine (Burtin and Michael, 1997), the free polyamine fraction (putrescine, spermidine and spermine) (Bassie et al., 2008) and the conjugated forms of polyamines (Prabhavathi and Rajam, 2007). Modifications in the second pathway of polyamine synthesis also led to changes in the polyamine content. Arabidopsis plants overexpressing the arginase gene were characterized by unchanged amounts of polyamines, while lines with a silenced arginase gene had an elevated level of putrescine (Brauc et al., 2012). However, overexpression of human ornithine decarboxylase in transgenic rice affected the enzyme activity, leading to its increase, which was accompanied by a significant rise of polyamine content (Lepri et al., 2001). Spermidine and spermine are produced from putrescine by spermidine synthase and spermine synthase, respectively. In this process S-adenosylmethionine decarboxylase, which catalyzes the conversion of S-adenosyl methionine to S-adenosylmethioninamine, is also involved.

In higher plants, polyamines participate in the growth and development processes, such as root growth, somatic embryogenesis, flowering initiation, and flower and fruit development. Furthermore, polyamines take part in the adaptation to the environmental conditions through the increase in plant resistance to abiotic (injury, salinity, drought, temperature) and biotic (pathogen infections) stressors. Previous research showed that polyamines can play a role in the response to pathogens, but their mechanism of action is not clear. It is suggested that polyamines may act as elicitors through activation of serine/threonine kinases from the MAPK group and an increase in the gene expression typical for HR, which lead to local cell death (Hussain et al., 2011). Polyamines can also act as activators of their catalytic enzymes, diamine oxidase and polyamine oxidase, which serve as a source of hydrogen peroxide. It is subsequently used by peroxidases in the lignification of the cell wall or as a local signal to initiate programmed cell death. Additionally, hydrogen peroxide can directly restrict pathogen growth (Walters, 2003a). Also polyamines can be involved directly in strengthening cell walls as they are associated with pectin polysaccharides and they are believed to control lignin deposition and cell wall pH (D'orazi and Bagni, 1987; Angelini et al., 1993).

Polyamines can also cause stabilization or destabilization of DNA via binding interactions. It is commonly known that such interactions influence the function of transcription factors and gene expression. It was reported that the synthetic analog of putrescine reduces the level of methylation of pathogen DNA through the inhibition of cysteine DNA methylase, causing the restriction of pathogen growth (Walters, 1997).

Polyamines are essential for the growth and development of both plants and fungi, yet the role of polyamines during pathogen infection remains questionable. There is a doubt whether polyamines protect plants against infection or are a source of nutrients for fungal pathogens (Divon and Fluhr, 2007). It was reported that a decrease in the amount of polyamines produced by pathogens due to a mutation of the ornithine decarboxylase gene leads to a reduction in their capacity to infect plants and inhibits pathogen growth (Bailey et al., 2000). During pathogen infection a local increase in the polyamine content may occur, which is defined as the creation of so-called “green islands.” The presence of these areas, close to the site of infection, guarantees pathogens continuous access to the nutrients. The local increase in the polyamine content may not results from the changes in their biosynthesis or degradation, but changes in the polyamine fraction. It has been observed that in the “green islands,” the free polyamine content increases, whereas the level of polyamines conjugated to hydroxycinnamic acids decreases (Walters, 2000).

The aim of this study was to elucidate the polyamine synthesis pathway in flax and assess the changes in polyamines during *Fusarium* infection. In particular, we determined the changes in the expression levels of genes involved in the polyamine metabolism after infection and measured the content of the polyamines putrescine, spermidine, and spermine, together with their fractions (free, conjugated to phenolic acids or bound to the cell wall). In order to better understand the role of polyamine in the infection process, we compared results obtained from analysis of plants during infection by two pathogenic strains of *F. oxysporum* and *F. culmorum*, and the non-pathogenic strain *F. oxysporum* Fo47.

As far as we know, this is the first report describing genes involved in polyamine synthesis and their identity and content in flax plants. Also their involvement in response to the pathogen has not been studied previously. These findings not only provide the first characterization of polyamines in flax, but also pave the way for engineering of transgenic plants that are more resistant to these dangerous pathogens.

## Materials and methods

### Plant material and growth conditions

Flax seeds (*Linum usitatissimum* L., cv. Nike) were obtained from the Flax and Hemp Collection of the Institute of Natural Fibres in Poland. The pathogenic strains of *F. culmorum* and *F. oxysporum* were provided by the Plant Breeding and Acclimatization Institute, Poznan, Poland. The non-pathogenic strain *F. oxysporum* ATCC Number: MYA-1198 (Fo47) was purchased from the ATCC Company (USA).

Seed germination was carried out on Murashige and Skoog medium (MS medium; Sigma-Aldrich), pH 5.8, solidified with 0.8% agar and supplemented with 1% sucrose under the 16 h light (21°C), 8 h darkness (16°C) regime for 7 days on Petri dishes. For induction, 1-week-old seedlings on MS medium were transferred onto a PDA (potato dextrose agar) medium (Sigma-Aldrich), pH 5.7, with the pathogenic strain *F. oxysporum* or *F. culmorum* or the non-pathogenic strain *F. oxysporum* Fo47, or without any fungi. *F. oxysporum*, *F. culmorum* and Fo47 were grown in PDA medium, respectively, for 7, 4, and 5 days before use. After 6, 12, 24, and 48 h, the treated and non-treated seedlings were collected separately, frozen in liquid nitrogen, and stored in a deep freezer (−70°C) before use. The biological material was prepared in three independent replications.

### Identification of cDNA sequences of polyamine biosynthesis genes

The identification of unknown cDNA sequences of the genes in question from flax was done via homology alignments with the known gene sequences from other plant species. The primers were designed for the most homologous regions. The template for the PCR reaction was cDNA obtained via reverse transcription of mRNA isolated from 9-day old flax seedlings (control). The PCR product was analyzed by gel electrophoresis, cleaned using a DNA Gel-out Kit and finally cloned using a TOPO TA Cloning Kit (Invitrogen). The sequencing was done by Genomed S.A. The GenBank database (http://www.ncbi.nlm.nih.gov/blast/) was used to verify the obtained DNA sequences by comparing them with the flax genome (*Linum usitatissimum* cv. Bethune), to identify sequences of investigated genes and to search their homology with genes from other plants. The Clustal Omega software was used to prepare phylogenetic trees for all genes.

### Gene expression analysis

The mRNA level for the investigated genes was determined using real-time PCR. The total RNA was isolated using an Invisorb Spin Plant RNA Mini Kit (Invitrogen) following the manufacturer's protocol, and the RNA integrity was examined by gel electrophoresis on 1.5% (w/v) agarose containing 15% (v/v) formaldehyde. The remaining DNA was removed via DNase I (Invitrogen) treatment. Then, RNA was used as a template for cDNA synthesis using a High Capacity cDNA Reverse Transcription Kit (Applied Biosystems).

Real-time PCR reactions were carried out using a DyNAmo SYBR Green qPCR Kit (Thermo Scientific) on an Applied Biosystems StepOnePlus Real-Time PCR System. The reaction conditions were applied according to the kit manufacturer's instructions, with an annealing temperature fixed at 58°C. The specificity of the primers at this temperature was confirmed by analysing the products using the melting curve method. The primer sequences used for the real-time PCR reactions are presented in Table 1. The reactions were carried out in three replicates. The actin gene was used as a reference gene. The changes in transcript levels were presented as the relative quantification to the reference gene.

**Table 1:** **Primer sequences for real-time RT-PCR reactions**.

**Gene**	**Forward primer**	**Reverse primer**
ACT	5′ CCGGTGTTATGGTTGGAAT 3′	5′ TGTAGAAAGTGTGATGC CAAA 3′
ADC	5′ GAGGAGCTTGATTTGGTGA 3′	5′ CACGCTGAGAATCTGAGTT 3′
AIH	5′ GTCTACTGCAACTGATGCTAA 3′	5′ AGAAGCAGCAAGTCTTGT 3′
NCPAH	5′ AAGCACAAAGGGAGGAC 3′	5′ CTATGGAATTGTAATGAGCAT TGTT 3′
ARG	5′ CTGATGTTGGTGATGTCCC 3′	5′ CCTCCAAGTTTCTCAGATAC AG 3′
ODC	5′ CTGGATGAGTGTTTCTCAGG 3′	5′ GCAATTCATCGACCCGTA 3′
SPDS	5′ AAAGCAGTCCTGGTAGTTG 3′	5′ CGTGGATCTTCGAATCCG 3′
SPS	5′ GGCTATTCCTTCCTGTTGTC 3′	5′ GGCCACATAGGATTGTTAA AGTA 3′
DAO	5′ CAAAGTCGGCATGATCG 3′	5′ GGGTTTACGTTGACCAGAG 3′
PAO	5′ ATGGGAGGACGTTTGTAG 3′	5′ GCCAGAACACATTGTCGAA 3′

### Polyamine extraction and analysis by UPLC

The isolation and fractionation of polyamines was performed using a modified version of the method described by Imai et al. (2004). Briefly, 100 mg of ground flax seedlings were extracted with 1 ml of 4% (w/v) perchloric acid (PCA) for 1 h at 4°C with shaking. After centrifugation (14,000 rpm, 20 min, 4°C), the supernatant was preserved and the pellet was resuspended in 0.5 ml of 4% PCA. The supernatant contains free polyamines and polyamines conjugated to phenolic acids, and the pellet contains insoluble polyamines bound to cell wall components.

In order to convert the conjugated and bound forms into a free form, 0.5 ml of 6 N HCl were added to 0.5 ml of the supernatant and suspended pellet and hydrolyzed at 110°C for 20 h. After drying in a speed-vac at 70°C, the residues were re-dissolved in 0.5 ml of 4% PCA.

Subsequently, polyamine dansylation was performed to facilitate identification: 0.2 ml aliquots were added to 0.2 ml of saturated sodium carbonate and 0.4 ml of dansyl chloride (5 mg/ml in acetone). After brief vortexing, the mixture was incubated in darkness at 60°C for 1 h. Excess dansyl reagent was removed by adding 0.2 ml of 150 mg/ml proline. Dansylated polyamines were extracted by 0.5 ml of toluene, dried in a speed-vac concentrator and re-suspended in 0.2 ml of acetonitrile, and finally 5 μl were injected on the column.

The samples were analyzed using a Waters Acquity UPLC system with a 2996 PDA detector on an Acquity UPLC BEH C18, 2.1 × 50 mm, 1.7 μm column. The mobile phase was A = water and B = acetonitrile, in a gradient flow: 1 min at 60% A/40% B; to 2.5 min, a gradient to 40% A/60% B; to 4 min, a gradient to 25% A/75% B; to 5.5 min, a gradient to 0% A/100% B; and to 6.5 min a gradient to 60% A/40% B with a 0.4-ml/min flow rate. The peak integration was done at 366 nm.

The results were standardized with mixtures of dansylated polyamine standards (putrescine, spermidine, spermine). Conjugated polyamine content was calculated by subtracting free polyamine content from the total acid-soluble polyamine content.

### Evaluation of polyamines' impact on *Fusarium* growth

Pathogenic strains of *F. oxysporum* and *F. culmorum* were grown on PDA medium for 7 days before the experiment. Then, fragments of this PDA medium of the same size, ingrown with fungi, were transferred onto plates with PDA medium (control plates) and PDA medium supplemented with polyamines (putrescine, spermidine, or spermine) at three concentrations (3, 6, and 10 mM). After 2 days of incubation, the plates were photographed and densitometric analysis was performed using the Bio 1D program. The data are presented as the percentage of the area of fungal growth on the medium with polyamines related to the fungal growth on the control medium.

### Statistical analyses

All of the experiments were independently repeated at least three times. The results are presented as the averages of independent replicates ± standard deviation. Statistical analyses were performed using Statistica 7 software (StatSoft, USA). The significance of the differences between the means was determined using Student's *t*-test.

## Results

### Identification of flax genes involved in polyamine metabolism

The aim of this study was to investigate the involvement of polyamines in the response to infection of flax with the pathogenic strains of *F. culmorum* and *F. oxysporum* and the non-pathogenic strain *F. oxysporum* Fo47. At first, genes that participate in polyamine metabolism were identified. The partial gene sequence of arginine decarboxylase (acc. no. GU581034.1) was obtained previously during the screening of a flax subtractive library (Kostyn et al., 2012).

The remaining cDNA sequences encoding for the genes from polyamine metabolism were isolated by means of reverse transcription PCR with primers designed for the most homologous regions of the searched genes from other species, as this part of the experiment was performed before the flax genome was released. In order to verify the obtained sequences, they were compared with the flax genome (*Linum usitatissimum* cv. Bethanum) sequenced by Wang and co-workers (Wang et al., 2012). As a result, the whole DNA sequences of investigated genes were identified. The comparison of obtained genes sequences—arginine decarboxylase, agmatine iminohydrolase (acc. no. JN191649.1), N-carbamoylputrescine amidohydrolase (acc. no. JN191648.1), arginase (acc. no. JN191650.1), ornithine decarboxylase, spermidine synthase (acc. no. JN191651.1), spermine synthase, diamine oxidase, and polyamine oxidase—with other sequences from the GenBank database (http://www.ncbi.nlm.nih.gov/blast/) revealed that flax polyamine genes show the highest homology (81–71% identity) to *Populus trichocarpa* (Supplementary Table 1). Additionally, phylogenetic analyses of polyamine-related gene families using sequences from different plant genomes were prepared (Supplementary Figure 1).

### Treatment with pathogenic and non-pathogenic *Fusarium* strains increases the transcript levels of genes involved in polyamine biosynthesis

We were primarily interested in the role of polyamines in the first stage of infection with pathogenic *Fusarium* strains, when no morphological changes in flax are observed but the pathogens are present in the plant tissues. Our previous research revealed that incubating plants with fungi for 48 h was optimal for tissue invasion (Wojtasik et al., 2011), so this period of time was used throughout this study. The presence of fungi in the plant tissue was assessed using PCR with two sets of primers for *Fusarium*-specific genes (data not shown). Additionally, three earlier time points (6, 12, and 24 h) were analyzed to investigate possible changes in polyamine metabolism in the early stage of infection before the fungus penetrates plant tissues. In order to correlate pathogen infection with polyamine metabolism, the transcript levels of polyamine genes after *Fusarium* infection were measured using real-time RT-PCR.

**Figure 2:**
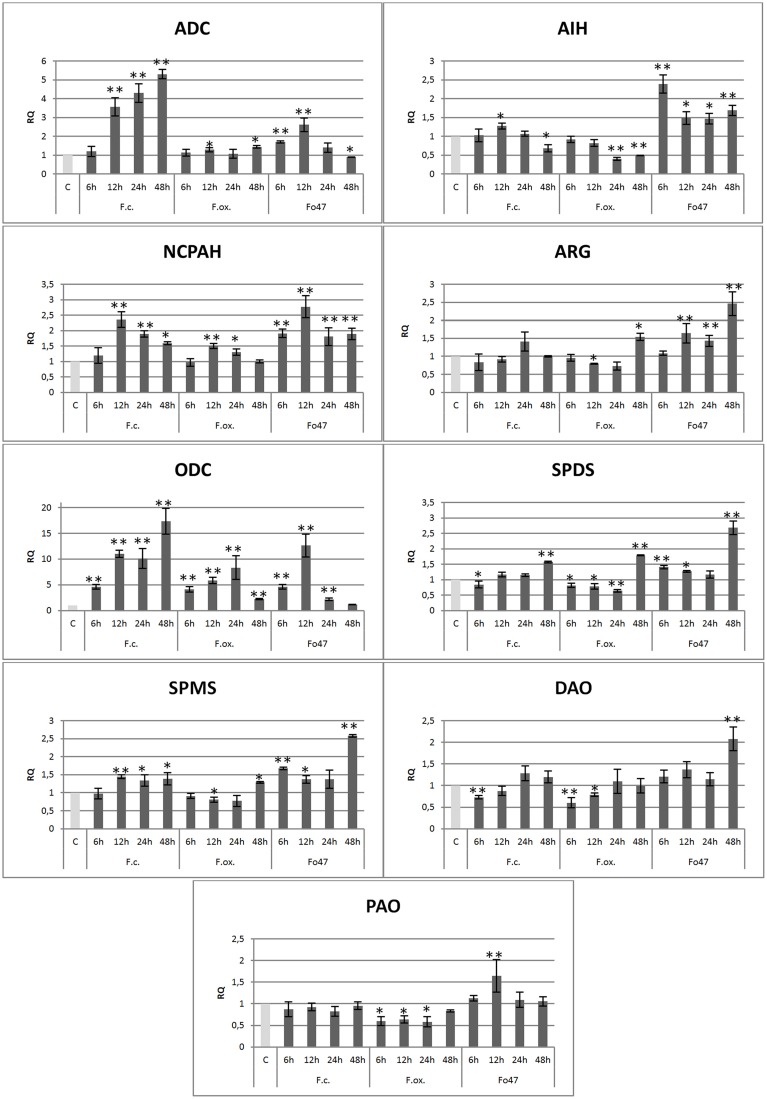
**Expression level of arginine decarboxylase (ADC), agmatine iminohydrolase (AIH), N-carbamoylputrescine amidase (NCPAH), arginase (ARG), ornithine decarboxylase (ODC), spermidine synthase (SPDS), spermine synthase (SPMS), diamine oxidase (DAO) and polyamine oxidase (PAO) genes in flax seedlings infected with pathogenic strains of *Fusarium culmorum* and *Fusarium oxysporum* and the non-pathogenic strain of *Fusarium oxysporum* (Fo47) at 6, 12, 24, and 48 h after inoculation**. The data were obtained from real-time RT-PCR analysis. Actin was used as a reference gene and the transcript levels were normalized to the control plant (C). The data represent the mean ± standard deviations from three independent experiments. The significance of the differences between the means was determined using Student's *t*-test (^*^P < 0.05, ^**^P < 0.01). RQ, relative quantity.

The mRNA levels of most polyamine biosynthesis genes were increased when compared to the control in flax seedlings co-incubated with *Fusarium* strains, whereas the transcript levels of polyamine degradation genes were not changed (Figure 2).

The biggest increases (up to 17-fold for *F. culmorum*, 8.4-fold for *F. oxysporum* and 12.5-fold for Fo47) were observed in the expression level of the ornithine decarboxylase gene, participating in one of the pathways of polyamine biosynthesis. A growth trend of the mRNA levels of the ODC gene depended on *Fusarium* strains, indicating the constant increase in time until 48 h of incubation for *F. culmorum*, growth to 24 h and then a decrease for *F. oxysporum*, and growth to 12 h and then a decrease for Fo47. The expression of the second gene of this pathway, encoding arginase, increased up to 1.5-fold only at 48 h after incubation with *F. oxysporum* and showed an increasing tendency from 12 to 48 h for Fo47.

The first gene of the alternative pathway generating putrescine, arginine decarboxylase, demonstrated similar behavior to the ODC gene. There was the same tendency in changes of mRNA levels, but the changes were lower (up to 5.3-fold after 48 h incubation with *F. culmorum*, up to 1.4-fold after 48 h incubation with *F. oxysporum* and up to 2.8-fold after 12 h incubation with Fo47). The expression of the next gene in the synthesis pathway, agmatine iminohydrolase, was reduced to 50% after *F. oxysporum* infection or initially was increased to 2.4-fold at 6 h incubation and finally was reduced at 48 h for Fo47. The gene encoding N-carbamoylputrescine amidohydrolase was found to be activated after infection with *Fusarium* strains and was characterized by the same trend (the highest points of mRNA level were at 12 h after incubation with fungi—up to 2.3-fold for *F. culmorum*, up to 1.5-fold for *F. oxysporum* and up to 2.8-fold for Fo47). Expression of the arginine decarboxylase gene increased 5.3-fold after *F. culmorum* and 1.4-fold after *F. oxysporum* infection.

The mRNA levels of the final genes of these pathways, encoding spermidine synthase and spermine synthase, were found to be significantly activated at 48 h after incubation with Fo47 (up to 2.6-fold). The rest of the changes in expression of genes participating in spermidine and spermine biosynthesis were inconsiderable and were in the range from a 1.4-fold increase to an 80% reduction in comparison with the control.

The infection of flax seedlings by *Fusarium* strains caused only slight changes in the expression levels of genes involved in polyamine degradation. The biggest observed alterations were the increase up to 2-fold in mRNA level of the diamine oxidase gene after 48 h co-incubation with Fo47 and up to 1.6-fold in mRNA level of the polyamine oxidase gene after 12 h co-incubation with Fo47. In the presence of *F. culmorum* only the expression of diamine oxidase genes was reduced to 80% of the control, whereas in the presence of *F. oxysporum*, expression of diamine oxidase and polyamine oxidase genes was reduced to 60% of the control.

### Exposure to fungi from *Fusarium* species increases the polyamine content in flax seedlings

**Figure 3:**
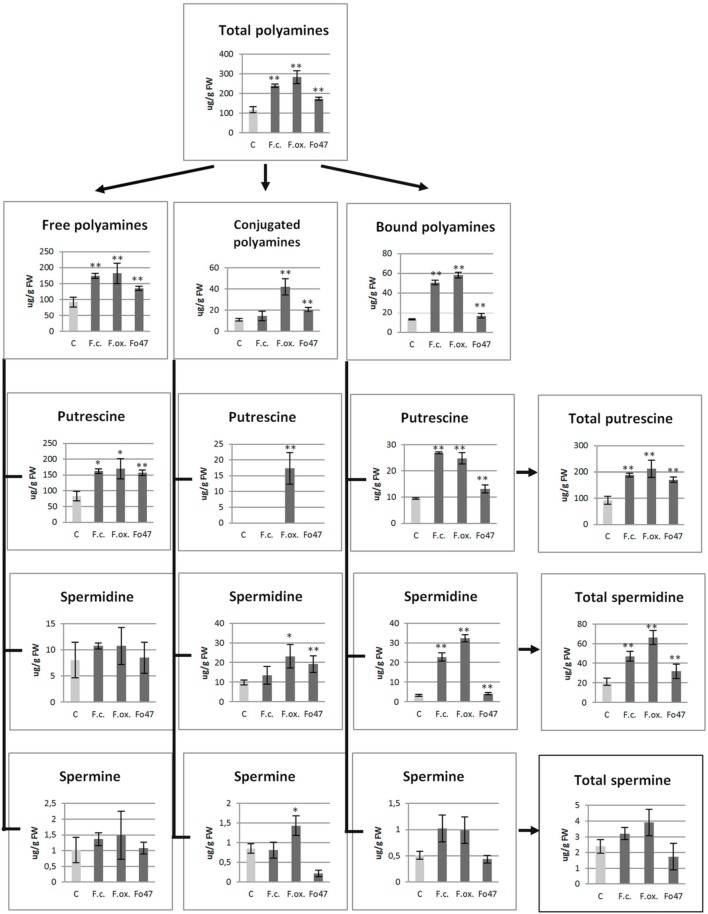
**Polyamine contents in flax seedlings after pathogenic strains of *Fusarium culmorum* and *Fusarium oxysporum* and the non-pathogenic strain of *Fusarium oxysporum* (Fo47) infection**. Quantities of total polyamines, free polyamines, soluble-conjugated polyamines and bound (insoluble-conjugated) polyamines, including putrescine, spermidine, and spermine, in flax seedlings after 48 h of *Fusarium* infection are presented in comparison with the values for the control plants (C). The data were obtained from UPLC analysis and represent the mean ± SD from four independent measurements. The significance of the differences between the means was determined using Student's *t*-test (^*^P < 0.05, ^**^P < 0.01).

The polyamines in flax are present mostly in free form, with only a small fraction present as conjugates and cell wall-bound form (Figure 3). Putrescine was determined to be the predominant polyamine in flax seedlings.

Even though mRNA levels started to increase for some genes as early as 6 h, significant changes in the polyamine content were observed in flax seedlings only after 48 h of co-incubation with pathogenic and non-pathogenic *Fusarium* strains (Figure 3). No significant differences were observed in the early hours of treatment; therefore these data are not shown. The total polyamine content was 2.5-, 2.0-, and 1.5-fold higher in flax seedlings treated with *F. oxysporum*, *F. culmorum*, and Fo47, respectively, in comparison with the non-treated flax seedlings.

Separation into the free, phenolic acid-conjugated and cell wall-bound fractions revealed the highest increase in the cell wall-bound fraction (up to 4.4-fold for *F. oxysporum* infection, up to 3.8-fold for *F. culmorum* infection and up to 1.9-fold for Fo47 treatment) as well as a large increase in the free polyamine fraction (up to 2-fold for *F. oxysporum* and *F. culmorum* and up to 1.9-fold for Fo47). Only *F. oxysporum* (*F. oxysporum* and Fo47) infection caused a significant increase (up to 4-fold and up to 1.3-fold, respectively) in the conjugated polyamine content.

In conclusion, the pathogen treatment significantly contributed to the increase in the total content of putrescine, spermidine, and spermine in all three polyamine fractions, and the changes in putrescine and spermidine contents were statistically significant. The contents of particular polyamine (putrescine, spermidine, and spermine) fractions are presented in Figure 3. It is very interesting to note that the cell wall-bound spermidine content was 7-fold higher after *F. culmorum* infection and 10-fold higher after *F. oxysporum* infection, while only 3-fold and 2-fold changes were observed, respectively, for bound putrescine and spermine. However, non-pathogenic Fo47 caused only slight changes in the cell wall bound polyamines (1.4-fold increase of putrescine and 1.3-fold increase of spermidine). The total content of putrescine (up to 2-fold higher) for flax seedlings treated with *Fusarium* strains resulted from higher levels of the free and cell wall polyamines, whereas the increase in total spermidine content (up to 2-fold for *F. culmorum*, 3-fold for *F. oxysporum* and 1.5-fold for Fo47) resulted from the increased level of the conjugated and cell wall bound spermidine.

### Free polyamines can restrict the growth of pathogenic *F. culmorum* and *F. oxysporum* in *in vitro* experiment

**Figure 4:**
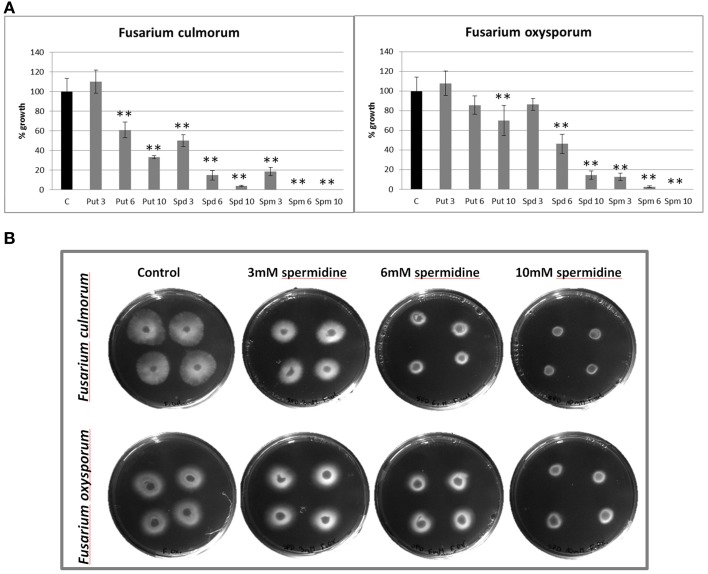
**The influence of exogenously applied polyamines on *Fusarium culmorum* and *Fusarium oxysporum* growth. (A)** Areas of *F. culmorum* and *F. oxysporum* growth after exposure to polyamines (putrescine, spermidine, and spermine) at three concentrations (3, 6, and 10 mM) were estimated by densitometric analysis of test plates in relation to control plates. The data represent the mean ± standard deviations from three independent experiments. The significance of the differences between the means was determined using Student's *t*-test (^*^P < 0.05, ^**^P < 0.01). putrescine: 3 mM (0.27 mg/ml), 6 mM (0.53 mg/ml), and 10 mM (0.89 mg/ml); spermidine: 3 mM (0.43 mg/ml), 6 mM (0.87 mg/ml), and 10 mM (1.45 mg/ml); spermine: 3 mM (0.61 mg/ml), 6 mM (1.21 mg/ml), and 10 mM (2.02 mg/ml). C, control; Put, putrescine; Spd, spermidine; Spm, spermine. **(B)** Growth of *F. culmorum* and *F. oxysporum* on plates with PDA medium (control) and PDA medium containing spermidine at three concentrations: 3 mM (0.43 mg/ml), 6 mM (0.87 mg/ml), and 10 mM (1.45 mg/ml).

In order to determine whether polyamines directly can affect *Fusarium* growth, we performed *in vitro* experiments where the fungal growth medium was supplemented with pure polyamine standards. The three investigated compounds (putrescine, spermidine, and spermine) reduced the growth of the pathogen in a dose-dependent manner (Figure 4A). The strongest antifungal activity was demonstrated by spermine, which showed a completely inhibitory effect on both *Fusarium* species at 10 mM concentration. Furthermore, 6 mM spermine stopped the spread of *F. culmorum*, but not the growth of *F. oxysporum*, although it was significantly restricted to 3% growth in relation to the control. For lower concentrations of the active agent, 1 mM solution stopped growth of 20% of *F. culmorum* in culture, while 0.1 mM concentration stopped growth of 8% of the pathogen colony (data not shown). Putrescine also reduced the growth of *F. culmorum.* and *F. oxysporum*, respectively, to 60 and 85% of the control at 6 mM concentration, and to 33 and 70% for 10 mM solution. Spermidine restricted *F. culmorum* and *F. oxysporum* growth, respectively, to 4 and 14% at 10 mM concentration, to 15 and 46% for 6 mM solution, and to 50 and 85% at 3 mM concentration. Figure 4B shows an example, where *Fusarium* growth was inhibited by addition of spermidine. The comparison between the two *Fusarium* species shows that *F. culmorum* is more sensitive to polyamines than *F. oxysporum*. We need to point out, however, that concentration used in *in vitro* experiment far exceed those found in flax plant.

## Discussion

In recent years, research concerning the involvement of polyamines in the plant response to pathogen infection has been focused on identifying the genes that regulate their biosynthesis pathways. Further study determined gene expression levels and the forms and amounts of the produced metabolites, and in particular any changes occurring in response to infection (Jimenez-Bremont et al., 2014; Montilla-Bascón et al., 2014).

In order to broaden the knowledge about the role of polyamines in the response to pathogen attack, we compared plant reactions to two pathogenic strains of *F. oxysporum* and *F. culmorum*, and a non-pathogenic strain of *F. oxysporum*.

In this study flax seedlings were infected with pathogenic *Fusarium* and the polyamine-related response to the first stage of infection was investigated. At the first stage, there were no visible morphological changes in the seedling phenotypes, however, the pathogen penetrates the plant tissues. The obtained data showed that *F. culmorum* and *F. oxysporum* infection of flax seedlings caused a significant increase in the mRNA levels of key genes from the polyamine biosynthesis pathways. However, it did not have an effect on the expression of genes involved in polyamine degradation.

It was justified to analyse *F. culmorum* and *F. oxysporum* infection individually because of different mechanisms of plant tissues penetration between these two pathogens. Additionally, the non-pathogenic *F. oxysporum* strain as a biological control was used to better understand the role of polyamines during pathogenic infection.

The highest changes were observed in the expression of ornithine decarboxylase (17-fold increase after *F. culmorum* infection and 2.2-fold increase after *F. oxysporum* infection), suggesting that the pathway that involves ornithine decarboxylation is the main route used for polyamine production in flax during infection. Furthermore, higher increase of ODC gene expression after treatment with *F. culmorum*, in comparison to *F. oxysporum* treatment, might indicate that the plant's response to the both fungi strains involves different mechanisms of defense. It was interesting that the second polyamine biosynthesis pathway was also strongly activated, noticing the 5.1-fold increase in the expression of arginine decarboxylase gene. On the other hand, after *F. oxysporum* infection the changes in the mRNA levels of putrescine synthesis genes were not so high and were observed after 48 h of co-incubation with the pathogen. However, the level of expression of spermidine synthase and spermine synthase was similar for both pathogens.

The changes in gene expression were accompanied by a 2-fold increase in the total polyamine content. The free fraction of polyamines has the highest percentage share in the total level of flax polyamines after fungal treatment, but the greatest differences in the contents of the compounds were noted for the bound fraction (4-fold increase after *F. culmorum* infection and 4.4-fold increase after *F. oxysporum* infection), indicating their great involvement in the plant defense response.

The comparison of pathogenic and non-pathogenic strains of *F. oxysporum* revealed differences in the tendency of changes in the expression levels of investigated genes coding proteins participating in polyamine biosynthesis. For almost all genes, the highest activation was observed after 6 h of co-incubation with non-pathogenic *F. oxysporum*, however, the changes in polyamine content were only noticeable after 48 h. The levels of expression of putrescine synthesis genes decreased in time, while for spermine and spermidine genes an increase was observed. What was unexpected was that, even though the changes in gene expression were higher after non-pathogenic *F. oxysporum* treatment, the polyamine content was still higher when compared to the control but it was relatively lower when compared to the pathogenic strain. The slight influence on content of investigated metabolites could be explained by the very transient increase of the gene expression and quick return to the initial level.

Studies aim to evaluate the involvement of polyamines in the plant response to pathogenic infections, however, they provide contradictory information as to the role of polyamines in repelling the pathogen attack. The data accumulated so far do not show a clear relationship between the polyamine levels and the plant infection state, so it might be suggested that the changes in the contents of polyamines depend on the type of pathogen or the means of infection. This unclear relationship of polyamine content and plant infection is perhaps the reason why some research suggests that plant polyamines are not involved in the defense mechanism but rather serve as a source of nutrition for fungi, although a negative influence of an increased polyamine level on the growth and development of fungi has been demonstrated.

In one study, it was noted that in wheat stalks, polyamine biosynthetic pathway genes were activated in the early stage of *F. graminearum* infection with highly accumulated putrescine in its free form (Gardiner et al., 2010). The free fractions of putrescine, spermine, and spermidine were also about accumulated in barley leaves infected with the powdery mildew fungus *Blumeria graminis* f. sp. *hordei*. Furthermore, this mildew infection also resulted in increased activities of the polyamine biosynthesis enzymes arginine decarboxylase (ADC), or ornithine decarboxylase (ODC) and s-adenosylmethionine decarboxylase (AdoMetDC) (Cowley and Walters, 2002).

However, in other studies, the polyamine content was reduced upon infection, such as in the case of tomato infected with *Rhizopus stolonifer*. Changes in the polyamine content were accompanied by a reduction in the activity of the enzymes ODC and ADC (Bakanashvili et al., 1987). The infection of tobacco leaves with *Peronospora tabacina*, *Erysiphe cichoracearum* and *Alternaria tenuis* also resulted in a decrease in the levels of free putrescine and spermidine (Edreva, 1997). The infection of sugarcane by the smut fungus *Ustilago scitaminea* caused a reduction in the level of free and conjugated forms of putrescine and spermidine (Legaz et al., 1998). No information is available in the literature about changes in the gene expression or the polyamine content in plants after non-pathogenic fungi strain treatment.

Also the data generated from transgenic plants give contradictory results. Transgenic tomato (*Solanum lycopersicum*) overexpressing yeast spermidine synthase accumulated higher levels of spermidine, but modified plants were more susceptible to *B. cinerea* than the wild-type plants (Nambeesan et al., 2012). However, sweet orange plants modified in the same way were characterized by enhanced plant tolerance to *Xanthomonas axonopodis* pv. *citri* (Fu and Liu, 2013). Furthermore, overexpression of the SPMS gene in *A. thaliana* resulted in an increased spermidine content and enhanced resistance against *Pseudomonas viridiflava* (Gonzalez et al., 2011). Similarly, overexpression of S-adenosylmethionine decarboxylase in plants caused increased tolerance to infection by *Pseudomonas syringae* and by *Hyaloperonospora arabidopsidis* (Marco et al., 2014).

One interesting aspect of the infection in flax is the significant increase in the polyamine contents, in particular in that of the cell wall-bound fraction. It was assumed that such accumulation of polyamines might contribute to the cell wall architecture rearrangements through their connection with cell wall components, maintaining wall coherency during stress responses (Lenucci et al., 2005).

There are no reports available on the role of bound polyamines in biotic stress, but their involvement in the abiotic stress response was reported (Minocha et al., 2014). In sugar beet roots, the bound fraction of putrescine increased significantly under salt stress. A similar, but smaller increase was found in tobacco roots. This indicates that bound putrescine contributes to protection against salt stress (Hajiboland, 2012). The same was detected for salt tolerance in soy bean roots (Wang et al., 2011). It has been demonstrated that polyamines are essential for maintaining the structural integrity of the developing plant cell wall by strengthening the links between cell wall components (Berta et al., 1997), so it could be speculated that they are involved in strengthening the physical barrier preventing pathogen infection.

Our preliminary data (unpublished) indicate changes in the cell wall composition and structure. In particular, an increase in the crystallinity index indicates more ordered and thus less reactive form of cellulose with lower absorbance of water and an increase in plasticity, ensuring greater cell wall stiffness, which could be helpful during pathogen infection. We can speculate that those changes could be, at least partially, initiated by changes in the accumulation of cell wall-bound polyamines.

One possibility is that polyamines, through the activation of catabolic enzymes, lead to the increased production of hydrogen peroxide, which is used during the infection in the lignification of the cell wall (Walters, 2003b). The level of polyamine oxidase expression was reduced in the flax seedlings infected with *F. oxysporum*, indicating a lack of involvement in the lignification process by polyamine at this stage of infection in this manner. How this binding is regulated and whether polyamines are bound to cell wall components on their own or as hydroxycinnamic acid amides needs to be investigated. The latter could be interesting as hydroxycinnamic acids are known for their antimicrobial and antifungal properties, and both di-p-coumaroyl-caffeoylspermidine and tri-p-coumaroylspermidine reduced the mycelial growth of the oat leaf stripe pathogen *Pyrenophora avenae* and also reduced the infective ability of powdery mildew (*Blumeria graminis f.* sp. *hordei*) (Walters et al., 2001).

Our other experiments show that free polyamines can have a direct inhibitory effect on fungal growth. However, the free polyamines were effective at concentrations far exceeding those found in flax seedlings. It is possible that conjugated forms of polyamines can be much more effective, and we do not know how the cell wall bound polyamines can affect fungal growth as there is no literature information about this fraction involvement in a pathogen response. We can only speculate that in plants, particularly in the cell wall the local concentrations of polyamines can be sufficient to inhibit spread of the disease. Such a local polyamine increase during infection can led to HR and local death of plant tissue. It is worth to note that effect of polyamine content could be species dependent.

The analysis of gene expression of the polyamine metabolism in flax infected with *F. culmorum* revealed two highly induced genes. The expression of the ornithine decarboxylase gene increased from the first stage of infection and was stronger than during *F. oxysporum* infection. In addition, at the late stage of infection, a strong increase in expression of the arginine decarboxylase gene was observed, which was not visible after *F. oxysporum* infection. The results indicate the activation of both pathways of putrescine synthesis but ornithine pathway is much more strongly activated. In the examples described earlier it can also be noted that, in some cases, in plants infected with various pathogens an increase in the expression of both ADC and ODC genes or only one of them might be observed.

In summary, we showed for the first time not only sequences of the flax polyamine genes but also established their expression patterns in the early stage of *Fusarium* infection in flax seedlings, indicating their involvement in the plant response to pathogens. The significant increase in the mRNA levels of polyamine biosynthesis genes was reflected in the increase in their amount. Although the role of polyamines in plant defense is still unclear, we can speculate that locally accumulated polyamines can directly inhibit pathogen growth, or through their connection with pectin and other cell wall compounds, they can reinforce the cell wall to restrict pathogen invasion. Furthermore, the defense mechanism activated in response to flax seedlings infected with *F. culmorum* was different than in the case of infection by *F. oxysporum*. This is due to the ability of fungi to damage the cell wall, and their method of spreading throughout the host. These findings open up the possibility for future genetic manipulation of flax in order to obtain new lines that are more resistant to *Fusarium* infection.

### Conflict of interest statement

The authors declare that the research was conducted in the absence of any commercial or financial relationships that could be construed as a potential conflict of interest.

## Supplementary material

Supplementary Figure 1 **Phylogenetic analyses of polyamine-related gene families participating in polyamine metabolism**.

Supplementary Table 1 **Accession numbers in GenBank and gene sequence homology for the partial sequences of polyamine biosynthesis genes**.
