# Supplementary material for: Polyamine metabolism in flax in response to treatment with pathogenic and non–pathogenic Fusarium strains
Source: Front Plant Sci. 2015 Apr 29;6:291. doi: 10.3389/fpls.2015.00291 (PMC4413726; doi:10.3389/fpls.2015.00291)
Supplement: Supplementary Figure 1 — Phylogenetic analyses of polyamine-related gene families participating in polyamine metabolism. [file DataSheet1.DOCX]

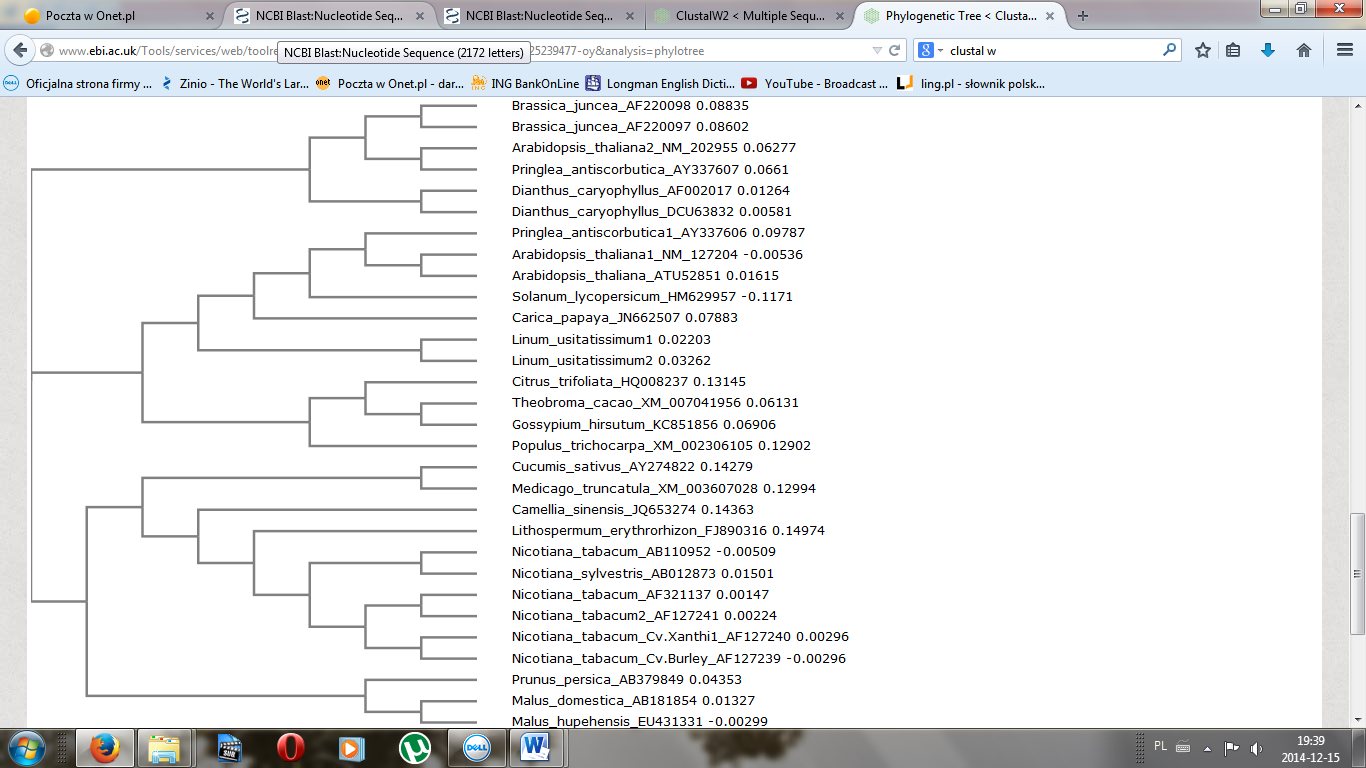


Arginine decarboxylase


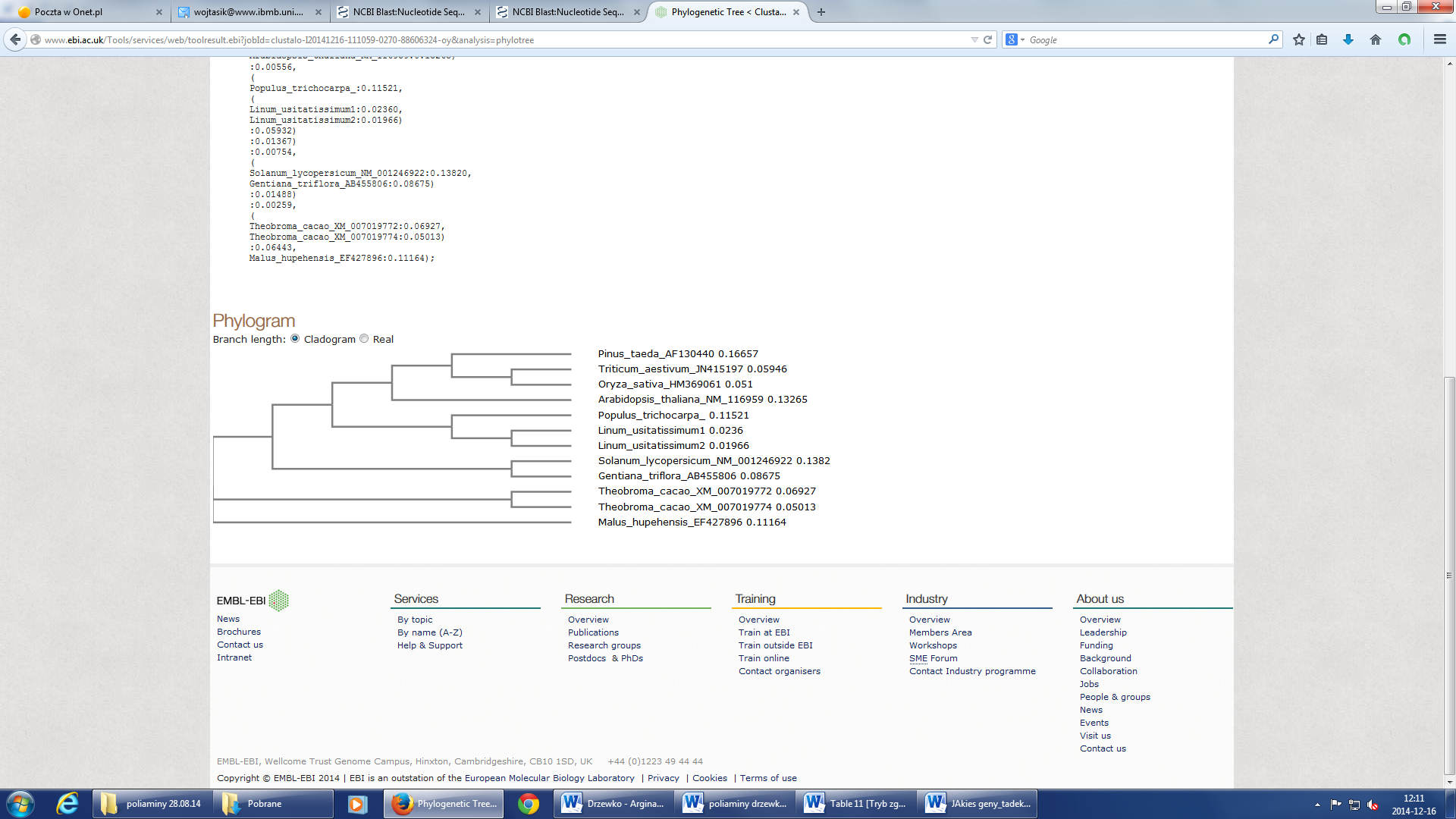


Arginase


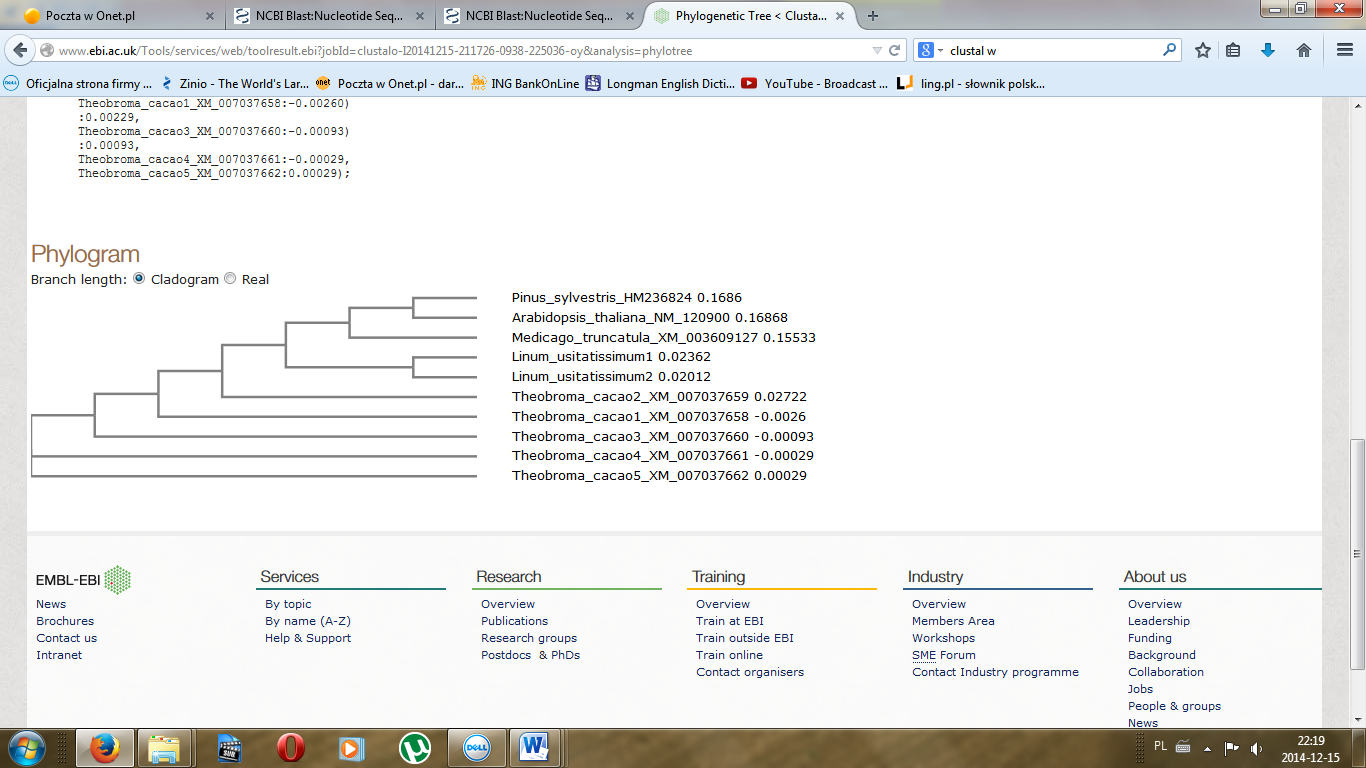


Agmatine iminohydrolase


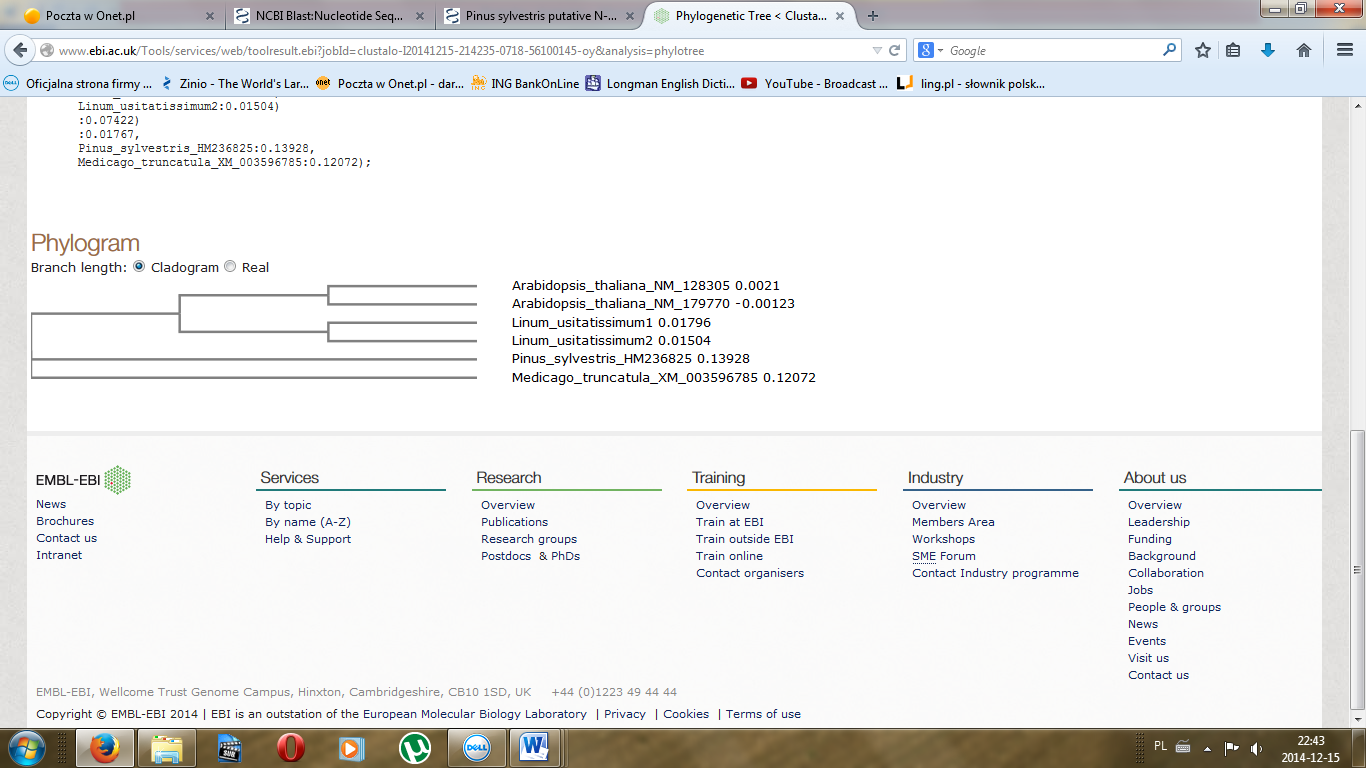


N-carbamoylputrescine amidohydrolase


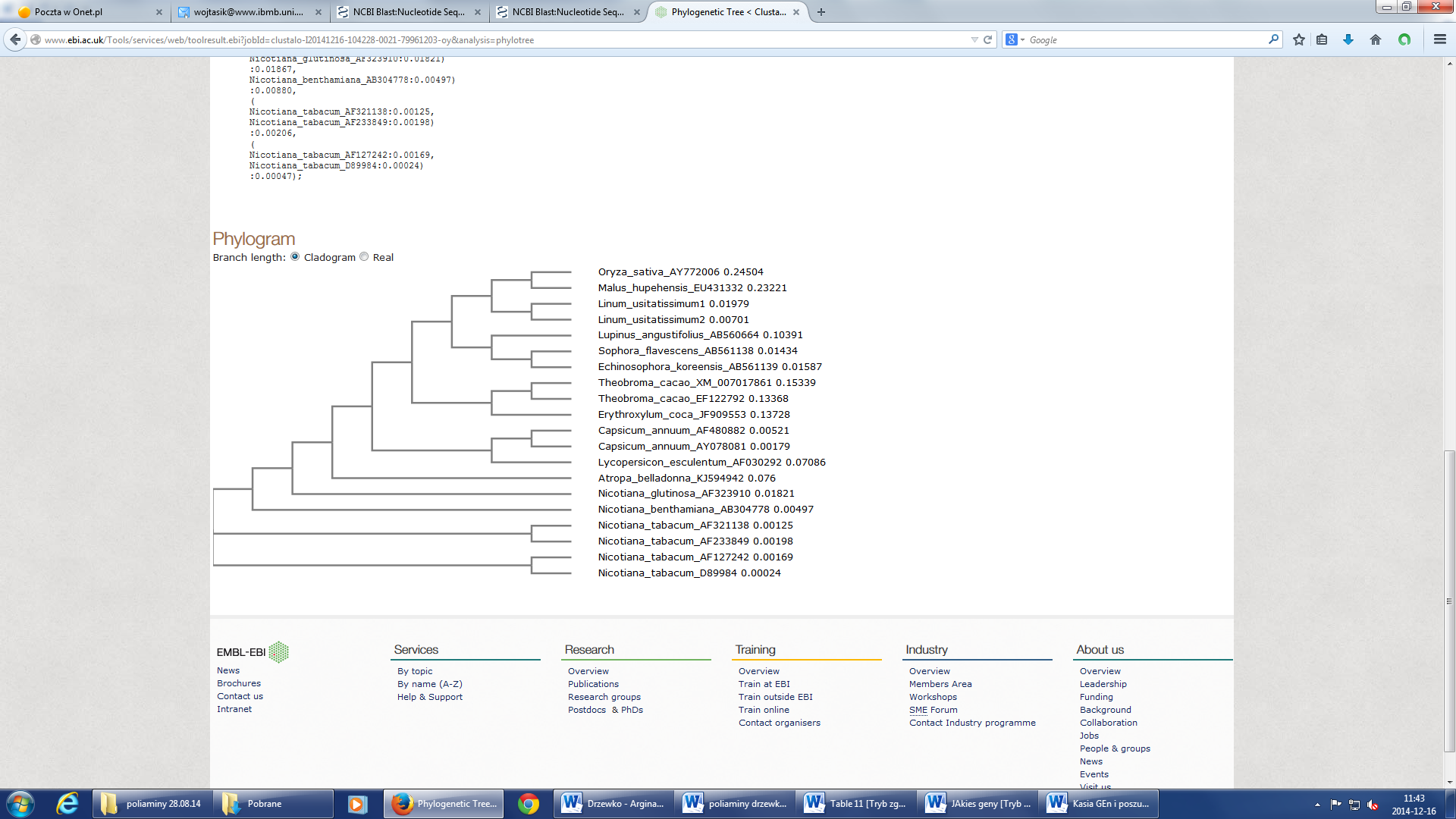


Ornithine decarboxylase


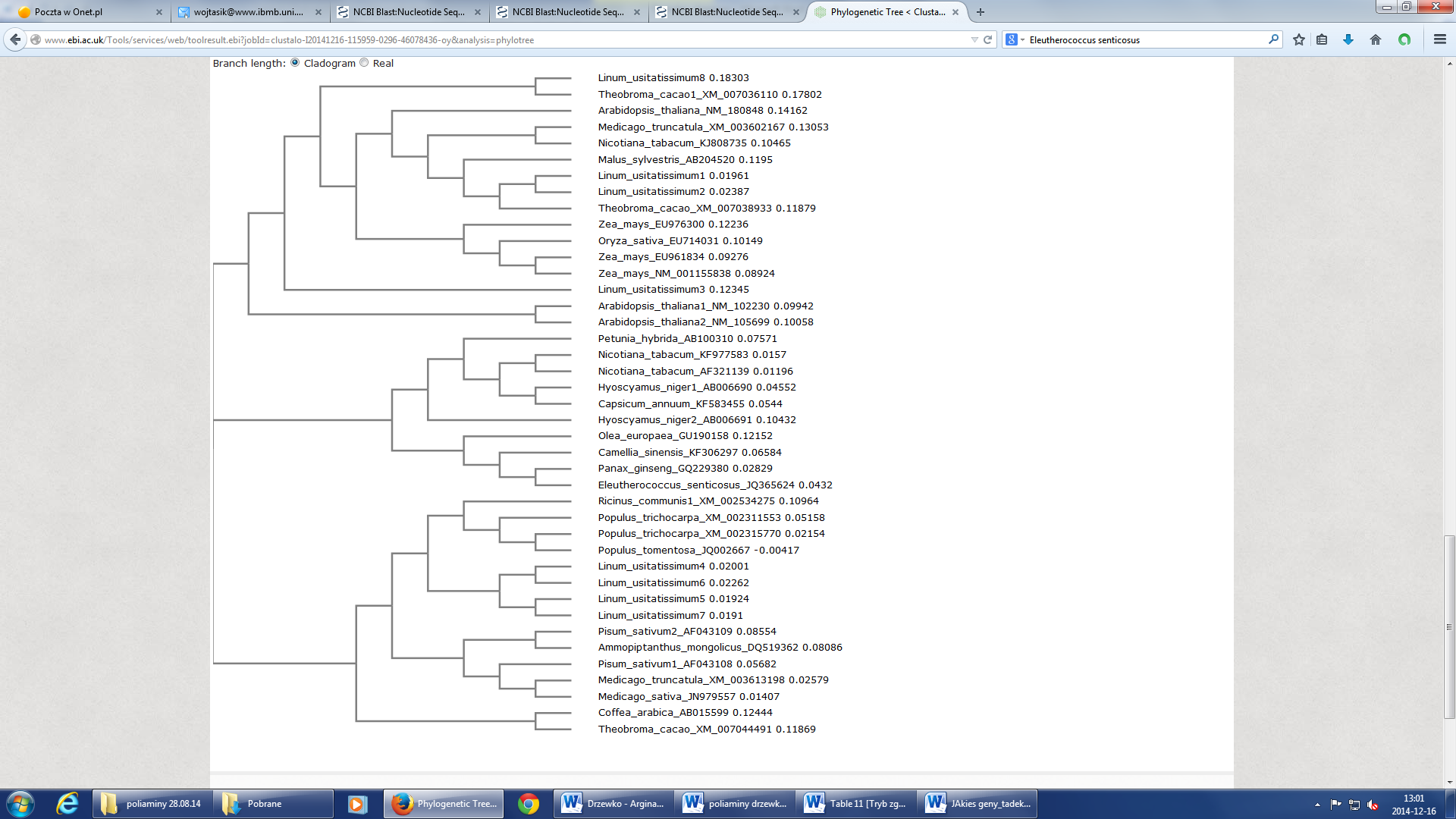


Spermidine synthase


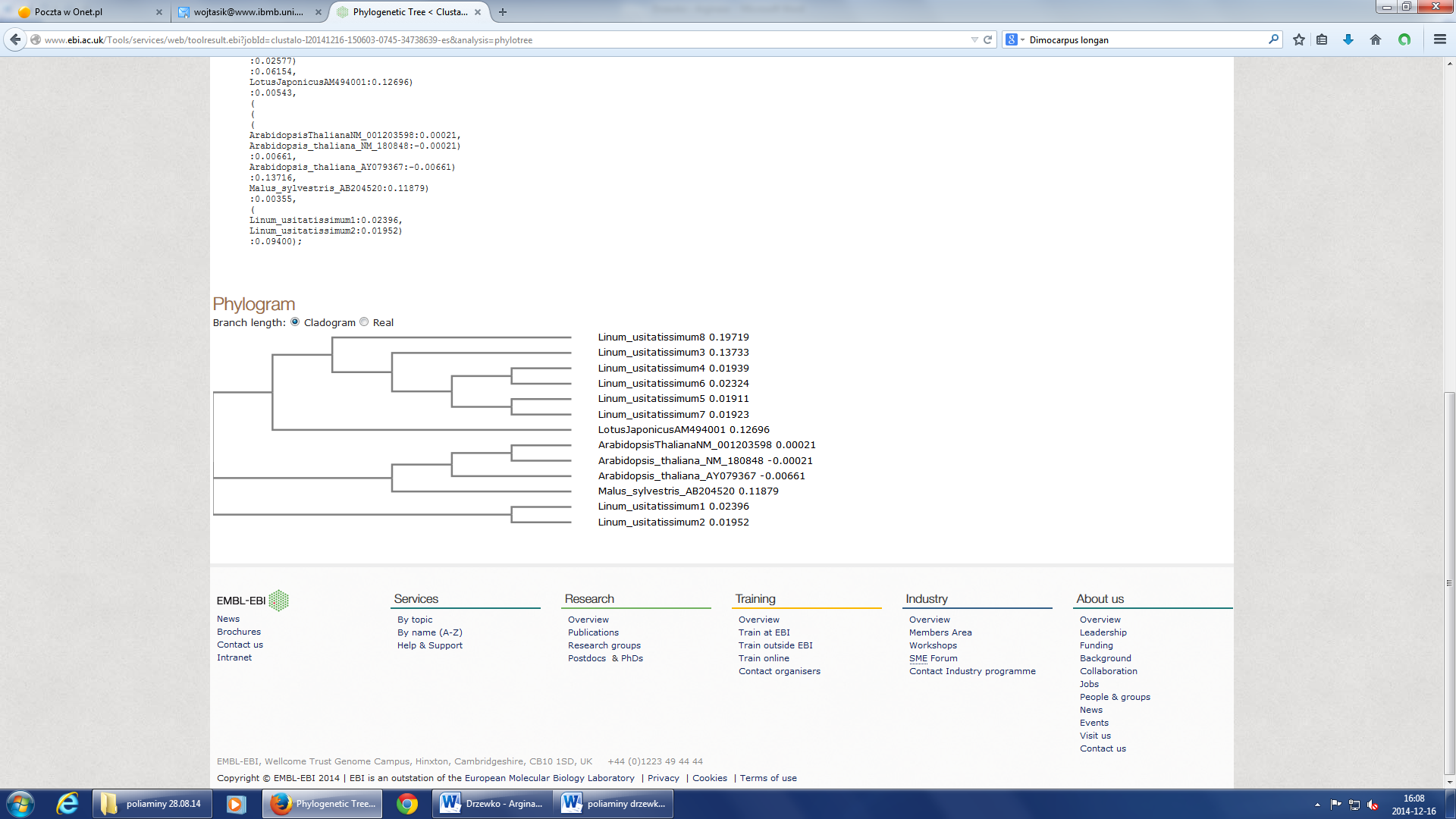


Spermine synthase


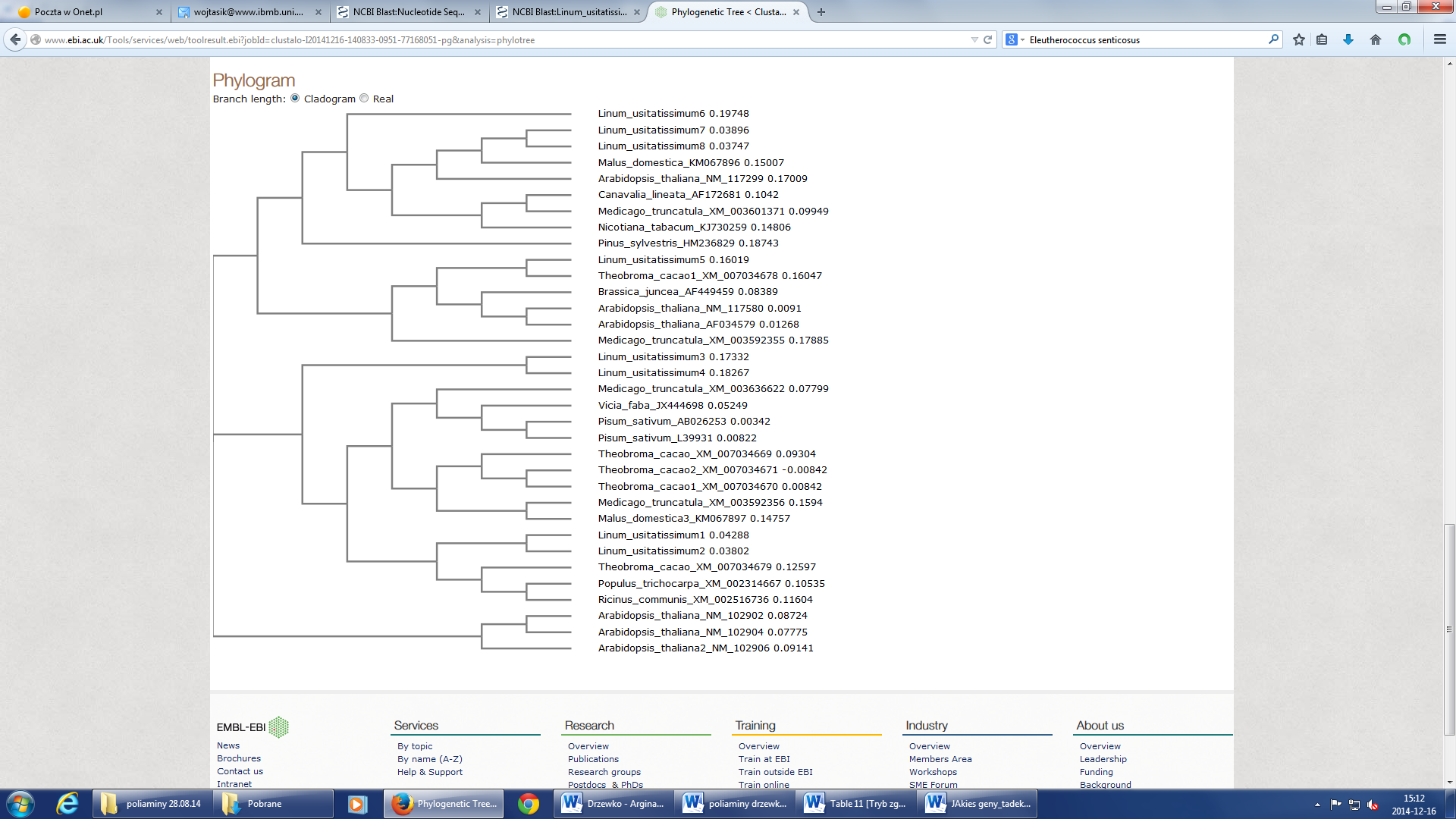


Diamine oxidase


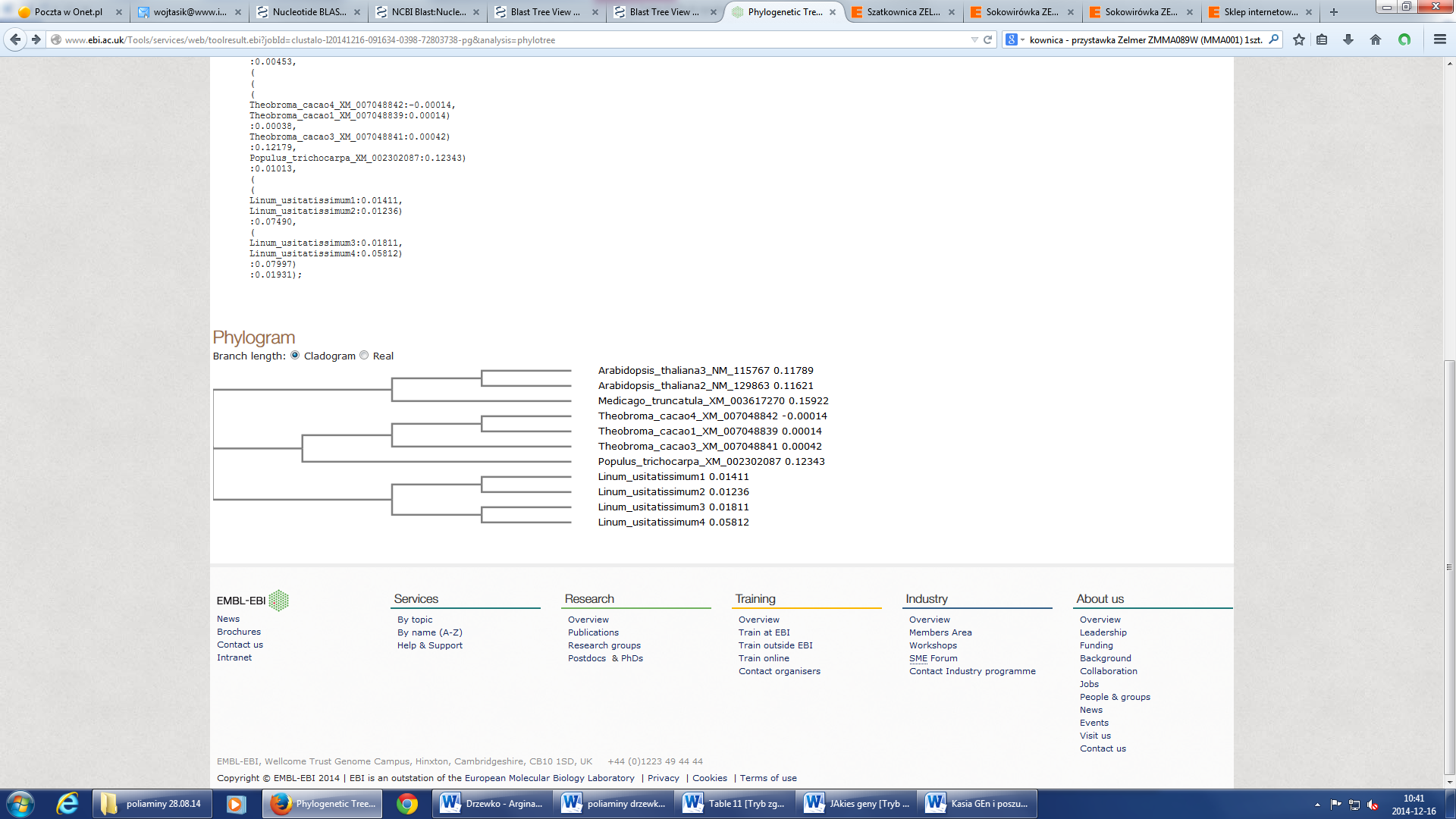


Polyamine oxidase

Supplementary figure 1
