# Supplementary material for: Polyamine metabolism in flax in response to treatment with pathogenic and non–pathogenic Fusarium strains
Source: Front Plant Sci. 2015 Apr 29;6:291. doi: 10.3389/fpls.2015.00291 (PMC4413726; doi:10.3389/fpls.2015.00291)
Supplement: Supplementary Table 1 — Accession numbers in GenBank and gene sequence homology for the partial sequences of polyamine biosynthesis genes. [file Table1.DOCX]

| **Gene description** | **GenBank**  **accession number** | **Identity (%)** |
| --- | --- | --- |
| arginine decarboxylase | [GU581034.1](http://www.ncbi.nlm.nih.gov/nucleotide/290760333?report=genbank&log$=nucltop&blast_rank=1&RID=AA4ZEGBP012) | Populus trichocarpa – 77%  Citrus trifoliata– 76% |
| agmatine iminohydrolase | JN191649.1 | Medicago truncatula– 78%  Theobroma cacao – 78% |
| N-carbamoylputrescine amidohydrolase | JN191648.1 | Arabidopsis thaliana– 79%  Medicago truncatula– 77% |
| arginase | JN191650.1 | Populus trichocarpa – 81%  Malus hupehensis – 79% |
| spermidine synthase | JN191651.1 | Populus trichocarpa – 79%  Theobroma cacao– 79% |
| spermine synthase |  | Malus sylvestris – 76%  Arabidopsis thaliana – 74% |
| ornithine decarboxylase |  | Theobroma cacao– 68%  Erythroxylum coca – 67% |
| diamine oxidase |  | Populus trichocarpa– 71%  Theobroma cacao – 70% |
| polyamine oxidase |  | Medicago truncatula – 74%  Theobroma cacao – 74% |

Supplementary table 1
